# Supplementary material for: A Laboratory-Developed Assay for the Simultaneous Detection of Aspergillus fumigatus and Pneumocystis jirovecii Pulmonary Pathogens
Source: J Fungi (Basel). 2025 Apr 2;11(4):280. doi: 10.3390/jof11040280 (PMC12028655; doi:10.3390/jof11040280)

Figure S2. Real-time PCR amplification curves for the ROX channel during optimization of the PJ-LDA. Various concentrations of $MgCl_2$ (a), KCl (b), primers (c), and probes (d) were tested in the probe-primer reconstitution mix. Images were generated using Hologic Open Access software (version 2.1.2.1). PJ, *Pneumocystis jirovecii*. LDA, laboratory-developed assay.

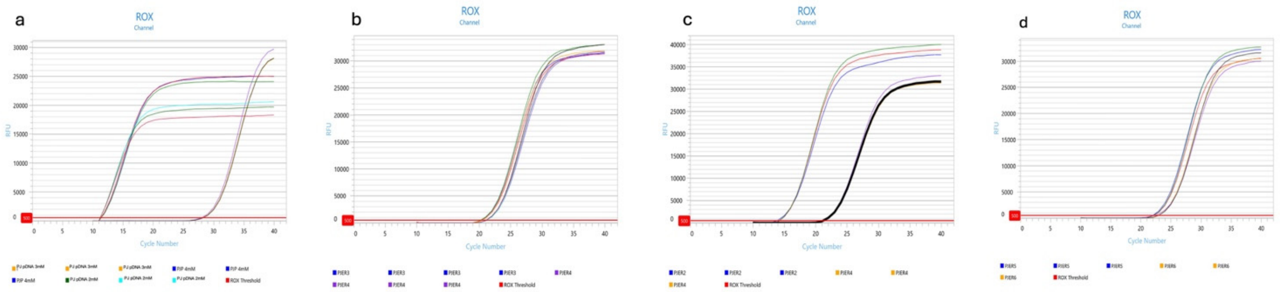

Supplement: Supplementary file 1 [file jof-11-00280-s001.zip › Figure S2.pdf]
