# Supplementary material for: A Laboratory-Developed Assay for the Simultaneous Detection of Aspergillus fumigatus and Pneumocystis jirovecii Pulmonary Pathogens
Source: J Fungi (Basel). 2025 Apr 2;11(4):280. doi: 10.3390/jof11040280 (PMC12028655; doi:10.3390/jof11040280)
Supplement: Supplementary file 1 [file jof-11-00280-s001.zip › Table S1.pdf]

Table S1. Microbial organisms used in analytical specificity testing of the laboratory-developed assay.

**Type**	**Source**	**Concentration (genome copies/reaction)**
Gram-negative bacteria (n = 16)		
Acinetobacter baumannii	ATCC 19606	10^5
Citrobacter koseri	ATCC 25408	10^5
Enterobacter cloacae	ATCC 13047	10^5
Enterococcus faecium	ATCC 700221	10^5
Enterococcus faecalis	ATCC 19433	10^5
Escherichia coli	ATCC 25922	10^5
Haemophilus influenzae	ATCC 33391	10^5
Klebsiella oxytoca	ATCC 13182	10^5
Klebsiella pneumoniae	ATCC 43816	10^5
Legionella pneumophila	ATCC 33152	10^5
Moraxella catarrhalis	ATCC 25238	10^5
Morganella morganii	ATCC 25830	10^5
Proteus mirabilis	ATCC 29906	10^5
Proteus vulgaris	ATCC 29905	10^5
Pseudomonas aeruginosa	ATCC 27853	10^5
Serratia marcescens	ATCC 13880	10^5
Gram-positive bacteria (n = 3)		
Staphylococcus aureus	ATCC 25923	10^5
Streptococcus pneumoniae	ATCC 49619	10^5
Streptococcus pyogenes	ATCC 19615	10^5
Fungi (n = 9)		
Aspergillus flavus	ATCC 12693	10^5
Aspergillus nidulans	ATCC 38163	10^5
Aspergillus niger	ATCC 16404	10^5
Aspergillus oryzae	ATCC 1011	10^5
Aspergillus terreus	ATCC 20542	10^5
Fusarium solani	ATCC 201839	10^5
Rhizopus oryzae	ATCC 20344	10^5
Candida albicans	ATCC 76615	10^5
Candida parapsilosis	ATCC 22019	10^5

DNA extracted from the listed American Type Culture Collection (ATCC) reference strains was spiked into bronchoalveolar lavage fluid and used at the specified concentrations for analytical specificity studies.
