# Supplementary material for: A Laboratory-Developed Assay for the Simultaneous Detection of Aspergillus fumigatus and Pneumocystis jirovecii Pulmonary Pathogens
Source: J Fungi (Basel). 2025 Apr 2;11(4):280. doi: 10.3390/jof11040280 (PMC12028655; doi:10.3390/jof11040280)
Supplement: Supplementary file 1 [file jof-11-00280-s001.zip › Table S2.pdf]

Table S2. Reagent concentrations tested for optimizing a real-time PCR-based LDA targeting *A. fumigatus* and *P. jirovecii*.

Reagent	Final concentration in the PCR reaction
$MgCl_2$	
$MgCl_2$, mM	2, 3, 4
KCl, mM	50
Tris, mM	12.5
Forward primer, μM	0.2
Reverse primer, μM	0.2
Probe, μM	0.1
KCl	
$MgCl_2$, mM	4
KCl, mM	50, 65
Tris, mM	12.5
Forward primer, μM	0.2
Reverse primer, μM	0.2
Probe, μM	0.1
Primers	
$MgCl_2$, mM	4
KCl, mM	50
Tris, mM	12.5
Forward primer, μM	0.2, 0.4
Reverse primer, μM	0.2, 0.4
Probe, μM	0.1
Probe	
$MgCl_2$, mM	4
KCl, mM	50
Tris, mM	12.5
Forward primer, μM	0.2
Reverse primer, μM	0.2
Probe, μM	0.1, 0.2

Reagents were optimized in the following order: $MgCl_2$, KCl, primers, and probes, for each of the two LDA targets. Optimization was performed independently in separate PPR mix tubes. The concentrations listed represent the final concentrations used in the PCR reaction. All reagents were initially prepared at 1.25× the final concentrations indicated.

Abbreviations: LDA, laboratory-development assay; PCR, polymerase chain reaction; PPR, primer-probe reconstitution mix.
